# *De novo* Transcriptome Assembly of Common Wild Rice (*Oryza rufipogon* Griff.) and Discovery of Drought-Response Genes in Root Tissue Based on Transcriptomic Data

**DOI:** 10.1371/journal.pone.0131455

**Published:** 2015-07-02

**Authors:** Xin-jie Tian, Yan Long, Jiao Wang, Jing-wen Zhang, Yan-yan Wang, Wei-min Li, Yu-fa Peng, Qian-hua Yuan, Xin-wu Pei

**Affiliations:** 1 College of Agriculture, Hainan Key Laboratory for Sustainable Utilization of Tropical Bioresources, Hainan University, Haikou, 570228, China; 2 Institute of Biotechnology, Chinese Academy of Agricultural Sciences, Beijing, 100081, China; 3 Institute of Plant Protection, Chinese Academy of Agricultural Sciences, Beijing, 100094, China; CSIR-National Botanical Research Institute, INDIA

## Abstract

**Background:**

The perennial *O*. *rufipogon* (common wild rice), which is considered to be the ancestor of Asian cultivated rice species, contains many useful genetic resources, including drought resistance genes. However, few studies have identified the drought resistance and tissue-specific genes in common wild rice.

**Results:**

In this study, transcriptome sequencing libraries were constructed, including drought-treated roots (DR) and control leaves (CL) and roots (CR). Using Illumina sequencing technology, we generated 16.75 million bases of high-quality sequence data for common wild rice and conducted de novo assembly and annotation of genes without prior genome information. These reads were assembled into 119,332 unigenes with an average length of 715 bp. A total of 88,813 distinct sequences (74.42% of unigenes) significantly matched known genes in the NCBI NT database. Differentially expressed gene (DEG) analysis showed that 3617 genes were up-regulated and 4171 genes were down-regulated in the CR library compared with the CL library. Among the DEGs, 535 genes were expressed in roots but not in shoots. A similar comparison between the DR and CR libraries showed that 1393 genes were up-regulated and 315 genes were down-regulated in the DR library compared with the CR library. Finally, 37 genes that were specifically expressed in roots were screened after comparing the DEGs identified in the above-described analyses.

**Conclusion:**

This study provides a transcriptome sequence resource for common wild rice plants and establishes a digital gene expression profile of wild rice plants under drought conditions using the assembled transcriptome data as a reference. Several tissue-specific and drought-stress-related candidate genes were identified, representing a fully characterized transcriptome and providing a valuable resource for genetic and genomic studies in plants.

## Introduction

Drought is one of the most common abiotic stresses that negatively influence plant growth, biomass production and crop yield [1]. Plants will activate a series of complicated regulatory mechanisms to deal with the unfavorable environment when experiencing drought [2–5]. For example, the regulation of abscisic acid (ABA) and other related transcription factors will work at the molecular and physiological and biochemical level [6–7]. On the other hand, at tissue and cellular level, plants respond to drought environment onset with morphological and cellular changes such as plasma membrane transform, growth retardation, stomatal closure, leaf wax increase, and the accumulation of osmoprotectants [8–12]. During the phenotypic and cell changes process, many genes that regulate metabolism at the physiological and biochemical level are highly expressed to enhance drought resistance [13–15]. Among these genes, several tissue-specific genes can induce morphological changes to address drought conditions. For example, *DRO1*, a deep-root gene, can induce rice roots to stretch into the deeper, wetter levels of soil to counteract the arid environment at the surface [16]. Overexpression of *OsNAC5* enlarges root diameter in rice plants, leading to enhanced drought tolerance [17], and *OsGL1-6* is involved in the accumulation of leaf cuticular wax and directly impacts drought resistance in rice [18]. With progress in molecular biology techniques, increasing numbers of genes related to drought have been isolated and identified.

As an important constituent part for plant, root systems have important biological roles in plants’ life cycle. It could anchor the plant, absorb water, inorganic salts and other nutrients, transferring hormones [19]. In recent years, more and more researchers focus on root system researches, including the growth and development of plant roots under drought stress [20]. When plants meet drought stress, responses occur in two different levels. At the morphological level, deep roots can absorb more water and nutrients; and at the molecular level, drought response genes in roots start to express as roots are among the primary sites for stress signal perception [21]. In previous reports, the responses of rice cultivar roots to drought, potassium stress, essential nutrients, structure, heavy metals and plant development have been analyzed on the transcriptomic level [22–26]. In addition, the second generation sequencing technology has been applied to identify stress-inducible transcripts in rice [27,28].

As the ancestor of cultivated rice, common wild rice which undergo a long history of artificial selection has a wide variety of excellent valuable mutated genes which have great potential value for rice breeding [29]. These plants grow in shallow water, small lakes and slow-flowing streams of eutrophication, and they possess the characters of disease and insect resistance and stress tolerance (resisting drought, high salt and low temperature), especially the developed ratoon systems.

Common wild rice grown in its natural habitat shows various resistances to biotic and abiotic stresses, as well as excellent genetic diversity. Previous studies have identified important genes controlling agronomic traits such as insect resistance, cold resistance, and high yield [30–32]. In addition, the *PROG1* gene, which is involved in rice domestication [33], and the *SH4* and *qSH1* genes, which control rice seed shattering [34], have been isolated. Certain genetic resources involved in drought resistance in common wild rice have been analyzed, including 12 drought-tolerance-related QTLs that were identified using introgression lines with the common wild rice Dongxiang accession as a donor [35]. However, the roots and tissue-specific gene expression in common wild rice has not been reported.

In the present study, common wild rice from the Gaozhou area of Guangdong province in China was used as material for studying root and drought resistance genes. The transcriptomes from different tissues of control and drought-treated common wild rice were sequenced using the Illumina paired-end sequencing technology, the sequencing data were assembled and annotated, and differentially expressed genes (DEGs) were identified. The research is essential and helpful to understand the transcript of the mechanism of different organizations drought in common wild rice, and it will be very useful for gene annotation and discovery and for genomic and transcriptomic assembly in common wild rice.

## Materials and Methods

### Plant Growth Conditions and Drought Stress Treatment

Common wild rice seeds were collected from Guangzhou Province in China. It was approved by supervision department of Guangdong wild rice protection. The seeds were sowed in Petri dishes at 35°C and then the plants were transferred to pots with water-culture after germination in a growth chamber. The seedlings were grown in four pots (30seedlings/pot) representing two treatments. The composition of the nutrient solution was as described by the International Rice Research Institute [36]. The plants were cultured under the following conditions: 50–70% relative humidity under short-day (8 h light/16 h dark) conditions at 30°C in the light and at 25°C at night.

After the seedlings grew for three weeks, one treatment was performed on the seedling sets using 15% PEG-6000 nutrient solution as the drought treatment for 24 h with the symptoms of wilting, and the second set of seedlings was used as an untreated control. Then, the root and shoot tissues of both control and treated seedlings were harvested and stored at −80°C in preparation for further assays.

### RNA Extraction and Transcriptome Sequencing

In this study, the shoot (CL) and root (CR) tissues of control plants and root tissue of the treated group (15% PEG-6000 stress for 24 h) (DR) were harvested for RNA extraction. RNA samples were extracted from five plants, and each sample was analyzed twice. Total RNA was extracted using Trizol reagent (Invitrogen, CA, USA) according to the manufacturer’s instructions, and the RNA quality was verified using the RNA Nano 6000 Assay Kit of the Agilent Bioanalyzer 2100 system (Agilent Technologies, CA, USA). The RNA Integrity Number (RIN) values for each of the samples were all greater than 8. For transcriptome sequencing libraries construction, NEBNext Ultra RNA Library Prep Kit for Illumina (NEB, USA) was used. First, mRNA was purified from the pooled total RNA using polyT oligo-attached magnetic beads and fragmented using an RNA fragmentation buffer. First-strand cDNA was synthesized using buffer, dNTPs, random hexamer primers and reverse transcriptase, and the second cDNA strand was synthesized using buffer, dNTPs, RNase H, and DNA polymerase I. Then the double-stranded cDNA was purified using the GeneJET PCR purification kit, then washed with EB buffer and submitted to end repair and single nucleotide adenine addition. Finally, adaptors were added to the fragments, and then were purified with AMPure XP beads and enriched by PCR amplification. After cluster generation, the sequencing libraries were sequenced by Illumina HiSeq 2000 platform.

### Data Filtering and De Novo Assembly

Raw sequence data generated from Illumina HiSeq 2000 platform were treated with following steps. First, remove adapters that were added for reverse transcription and sequencing, then sequences containing too many unknown bases (>10%) and low-quality bases (>50% of the bases with a quality score ≤5) were removed. After obtaining clean data for each of the six transcriptome libraries, the clean data were then mixed and assembly was done by using the Trinity software. The parameters were with min_kmer cov set to 2 by default and all other parameters set to the default settings [37]. All of the raw data was submitted to the database with the code Bioproject: SUX1023710 and BioSample: SRS933410.

### Unigene Annotation and Functional Classification

For unigene functional annotation, all the assembled unique gene sequences were searched against the NR (http://www.ncbi.nlm.nih.gov/), PFAM (HMMER 3.0 package, hmmscan) Swiss-Prot (http://www.expasy.ch/sprot/), KEGG (http://www.genome.jp/kegg/) and Eukaryotic orthologous groups (KOG) (http://www.ncbi.nlm.nih.gov/cog/) databases using BLASTx algorithms and against the NT database using the BLASTn algorithms, with a threshold of E<1.0E-5. KOG contradistinction algorithms with a threshold of E<1.0E-3 were used to determine the top 10 unigene alignment results. For the NR and PFAM annotations, the Blast2GO program was used to conduct GO annotation of unigenes, and the WEGO software was used to construct GO functional classification [38, 39].

### DGE Library Preparation, Sequencing and Mapping Analysis

Total RNAs from six samples, including shoot (CL) and root (CR) tissues of control plants and root tissue of the treated group (DR), two replicates each, were used for DGE library sequencing. For each sample, three micrograms of RNA was used for the DGE library construction. The process of the DGE sequencing library construction was same as those of the transcriptome sequencing libraries.

After getting the raw sequence data and performing the data-processing steps, the clean reads were then mapped to the assembly transcriptome reference sequences using the RSEM software [40]. Mismatches of no more than 2 bases were allowed in the alignment, and read counts for each gene were obtained from the mapping results.

### Identification of Differentially Expressed Unigenes

For calculating the gene expression level, the FPKM method was used [41], then the DEGs were screened by comparing the gene expression levels. The DESeq R package (1.10.1) [42] was used to analyze the DEGs between the two conditions. In order to control the false discovery rate, the resulting P values were adjusted using the Benjamini and Hochberg’s approach. In this study, genes with an adjusted P-value < 0.05 for CR vs CL with 1.5 times changes of gene expression and an adjusted P-value < 0.1 for DR vs CR with 1.5 times changes of gene expression were used for identifying the DEGs.

### Quantitative Real-Time PCR Analysis

To confirm the DEG results, quantitative real-time reverse transcription PCR (qRT-PCR) was performed on eighteen unigenes that were randomly chosen from the three libraries. qRT-PCR was implemented using the SYBR premix Ex Taq kit (TaKaRa, Dalian, China) on an ABI 7500 Real-Time System (Applied Biosystems). The relative expression value was calculated using the 2^-ΔΔCt^ method [43], with actin (*Os03g0718100*) as an internal control. The six known drought response genes, including *OsMYB2*, *SNAC1*, *OsNADPH-1*, *OsMAPK5 and OsAPX2* were also choosed for qRT-PCR analysis. In addition to these 18 unigenes, another four drought-induced unigenes were selected for expression analysis after different times of drought treatment. The gene-specific primers used in the qRT-PCR analysis are listed in S1 Table. The RNA pools used in the qRT-PCR analyses were extracted from three independent samples that were different from those used for RNA-seq. All reactions were performed using one biological sample and three technical replicates.

### Development and Detection of EST-SSR Markers

Besides screening DEG information, microsatellites distribution was also detected in the assemblied transcriptomes. The MISA software (http://pgrc.ipk-gatersleben.de/misa/misa.html) was used to identify microsatellites. EST-SSRs were considered to contain motifs consisting of one to six nucleotides.

Primers for each SSR were designed using Primer3 software (http://primer3.ut.ee).

## Results

### Illumina Paired-End Sequencing and De Novo Assembly

Six cDNA libraries were generated from the seedling stage (shoot and root of control, root-treated) of common wild rice using Illumina deep sequencing. After removing adapters and filtering the low-quality sequences, 6.42 million, 5.74 million, and 4.59 million clean 100-bp reads were generated for the control shoot (CL1, CL2), control root (CR1, CR2), and drought-treated root (DR1, DR2) libraries, respectively (Table 1). All of the high-quality reads from these six libraries were mixed to do transcriptome assembly by using the Trinity software [37]. Based on the overlapping information provided by the high-quality assembly, 201,352 transcripts, with a mean length of 1189 bp and an N50 of 2159 bp, were generated. After extracting the longest transcript for each gene to serve as the unigene, 119,332 unigenes were obtained. The average length was 715 bp, and approximately 38.88% of the unigenes were at least 500 bp long (Table 2, S1 Fig).

**Table 1:** Summary of data output quality of the various libraries.

Library	Raw Reads	Clean reads	Error(%)	Q20(%)	GC(%)
CL1	36,316,581	33,680,504	0.05	95.00	53.45
CL2	32,904,668	30,527,534	0.05	95.01	53.84
CR1	33,161,644	30,846,212	0.04	95.34	51.95
CR2	28,544,583	26,630,389	0.04	95.31	51.90
DR1	25,417,458	23,716,321	0.04	95.37	52.16
DR2	26,938,151	25,164,179	0.04	95.38	52.20

**Table 2:** Summary of the common wild rice transcriptome.

Category	Number				Total number	Mean length (bp)	N50 (bp)	Total nucleotides
	200-500bp	500-1kb	1k-2kb	>2kb				
Transcripts	84,613	39,012	38,705	39,022	201,352	1,189	2,159	239,341,036
Unigenes	72,936	24,321	13,563	8,512	119,332	715	1,147	85,352,510

### Functional Annotation and Classification of the Unigenes

**Fig 1:**
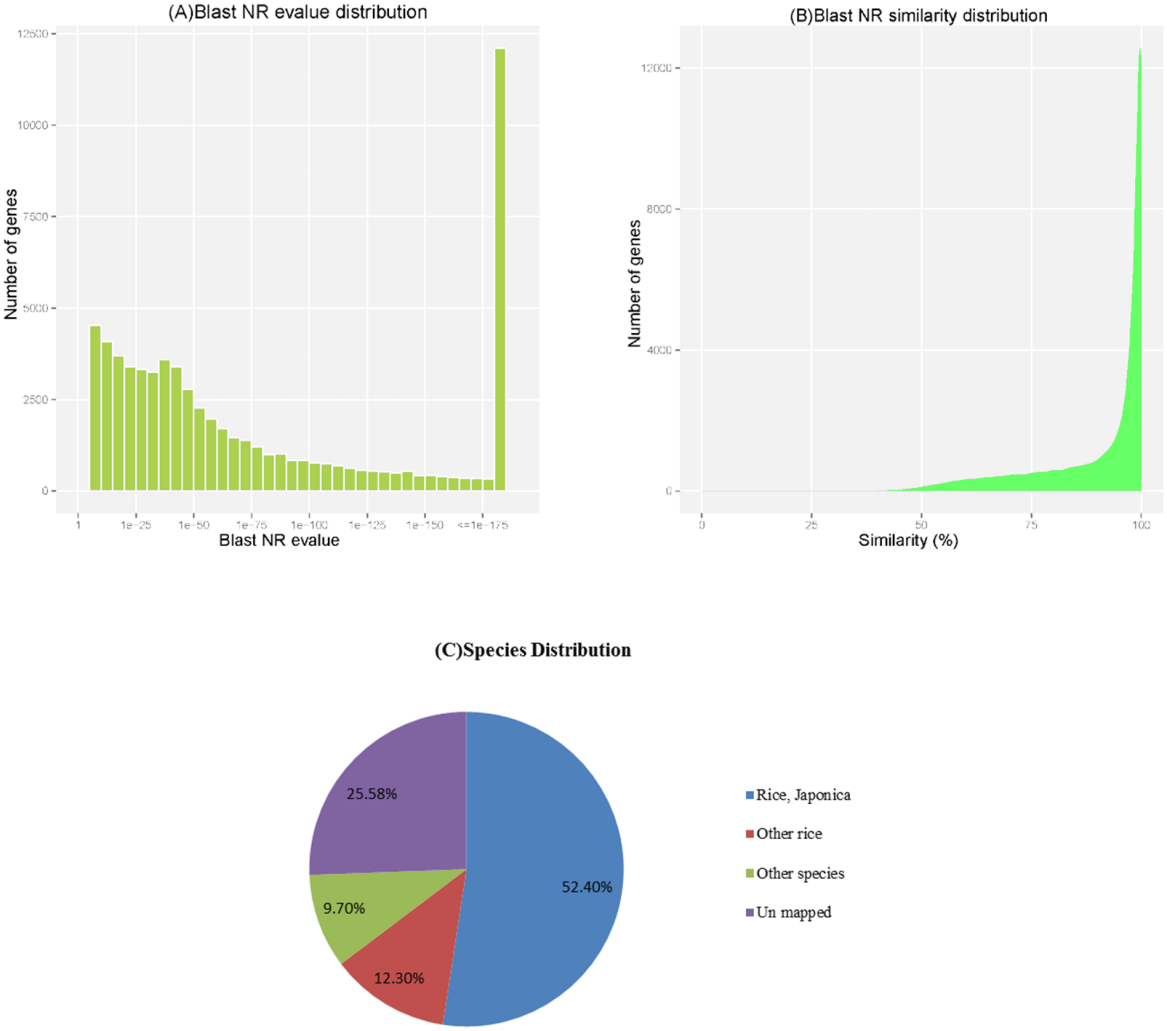
Characteristics of the BLAST matches of the transcriptome unigenes. (A) E-value and (B) Similarity distribution of the top BLAST hits for each unigene in the NR database, with a cut-off E-value of 1.0E-5. (C) Species distribution of the BLAST hits for each unigene in the NT database.

After getting the sequence of assembled unigenes, the sequences were searched against the SwissProt protein and NCBI non-redundant (NR) databases using the BLAST 2.2.28+ program (with E-value threshold of 1E-5). Of the 119,332 unique sequences, 111,171 unigenes (93.16%) had at least one significant match to an existing gene model. A total of 88,813 distinct sequences (74.42% of the unigenes) significantly matched known genes in the NT database (Table 3). Of the matched unigenes, approximately 18.87% (12424/65856) matched the NR database with an E value smaller than 1E-175 (Fig 1A), and approximately 71.71% (47226/65856) shared more than 90% similarity with an established sequence (Fig 1B). The majority of sequences (64.76%) were homologous to genes of *Oryza sativa*, and of those, 52.4% of the unigenes were best matched to sequences from *Oryza sativa* Japonica, followed by 12.3% from other *Oryza sativa* species and 9.7% from other species (Fig 1C). In the absence of matching to the NT database of 25.58% genes in 2572 genes have higher expression level, and 8161 genes were not matched to any database(S2 Table).

**Table 3:** Summary of functional annotation of the assembled unigenes.

Public database	Number of Unigenes	Percentage (%)
Annotated in NR	65,856	55.18
Annotated in NT	88,813	74.42
Annotated in KO	18,286	15.32
Annotated in SwissProt	38,097	31.92
Annotated in PFAM	42,906	35.95
Annotated in GO	55,318	46.35
Annotated in KOG	23,905	20.03
Annotated in all Databases	8400	7.03
Annotated in at least one Database	111,171	93.16
Total Unigenes	119,332	100

### Functional Classification by GO and KOG

**Fig 2:**
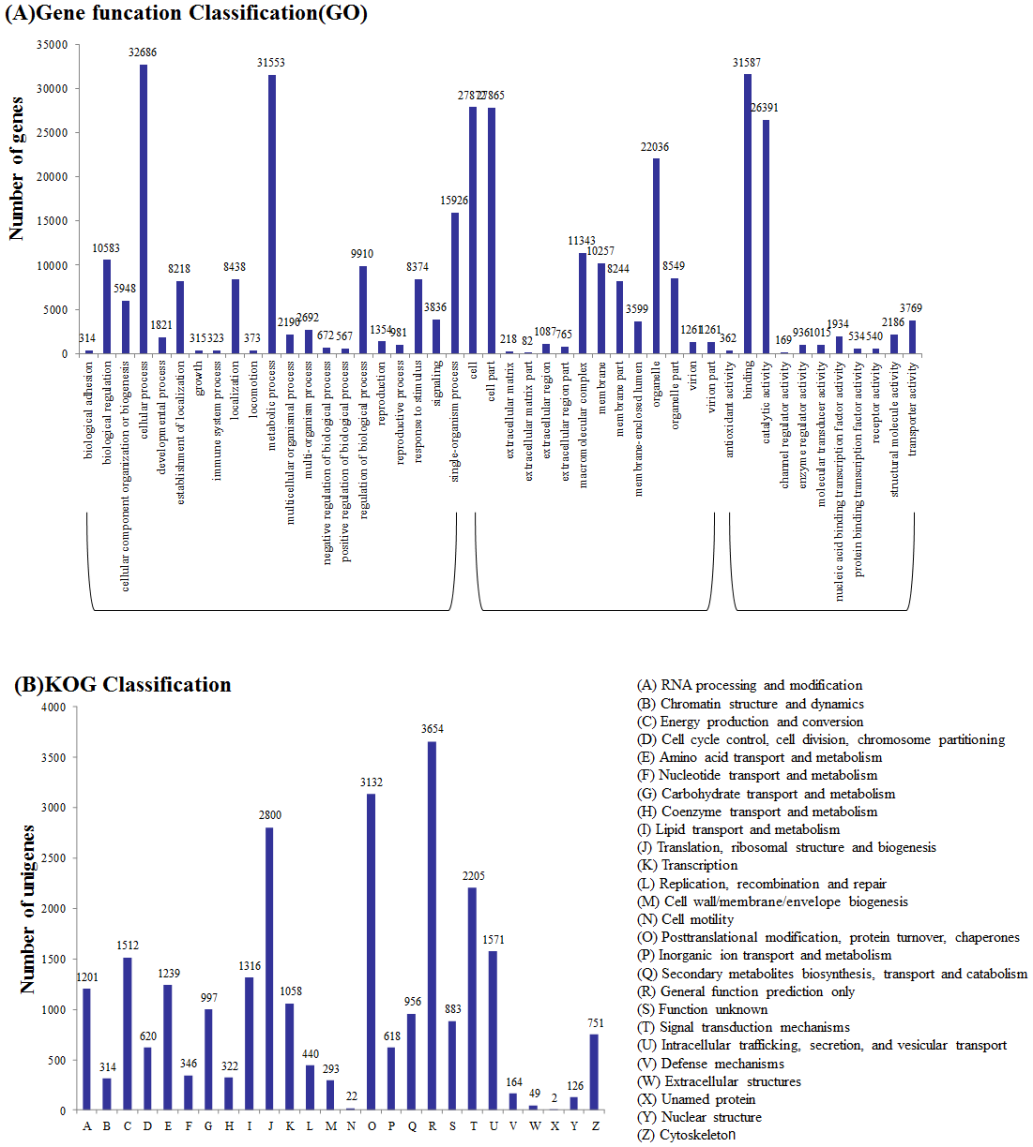
Functional classification of the assembled unigenes. (A) Comparison of Gene Ontology (GO) classifications of common wild rice. Unigene numbers assigned to the same GO terms are indicated above the bars; the x-axis indicates the subcategories, and the y-axis indicates the number of genes in a category. (B) Histogram of the Eukaryotic Orthologous Groups (KOG) classification.

Gene Ontology (GO) analysis was performed based on the NR annotation. Among the 65,856 annotated unigenes, 55,318 sequences were assigned to 56 GO functional groups terms (Table 2, S3 Table). Among the three main categories the biological process category was the most (147,154), following with the cellular components category (124,519) and molecular function category (69,458) (Fig 2A). Within the biological processes category, related to metabolic processes (21.44%) and cellular processes (22.21%) were the most enriched. Meanwhile, genes encoding binding proteins (45.42%) and proteins related to catalytic activity (37.99%) were enriched in the molecular function category. In the cellular components category, cell (22.38%), cell part (22.38%) and organelle (17.70%) were the largest proportion represented GO terms.

For KOG classifications, 23,905 sequences were assigned among the 65,856 NR annotated unigenes (Table 2). These sequences were assigned to 25 KOG categories, the cluster for general function prediction was the largest group (3654, 15.29%), others followed by posttranslational modification (3132, 13.10%), translation (2800, 11.71%), signal transduction (2205, 9.22%), intracellular trafficking and secretion (1571, 6.57%), and energy production and conversion (1512, 6.33%) (Fig 2B).

### Functional Classification by KEGG Pathway

**Fig 3:**
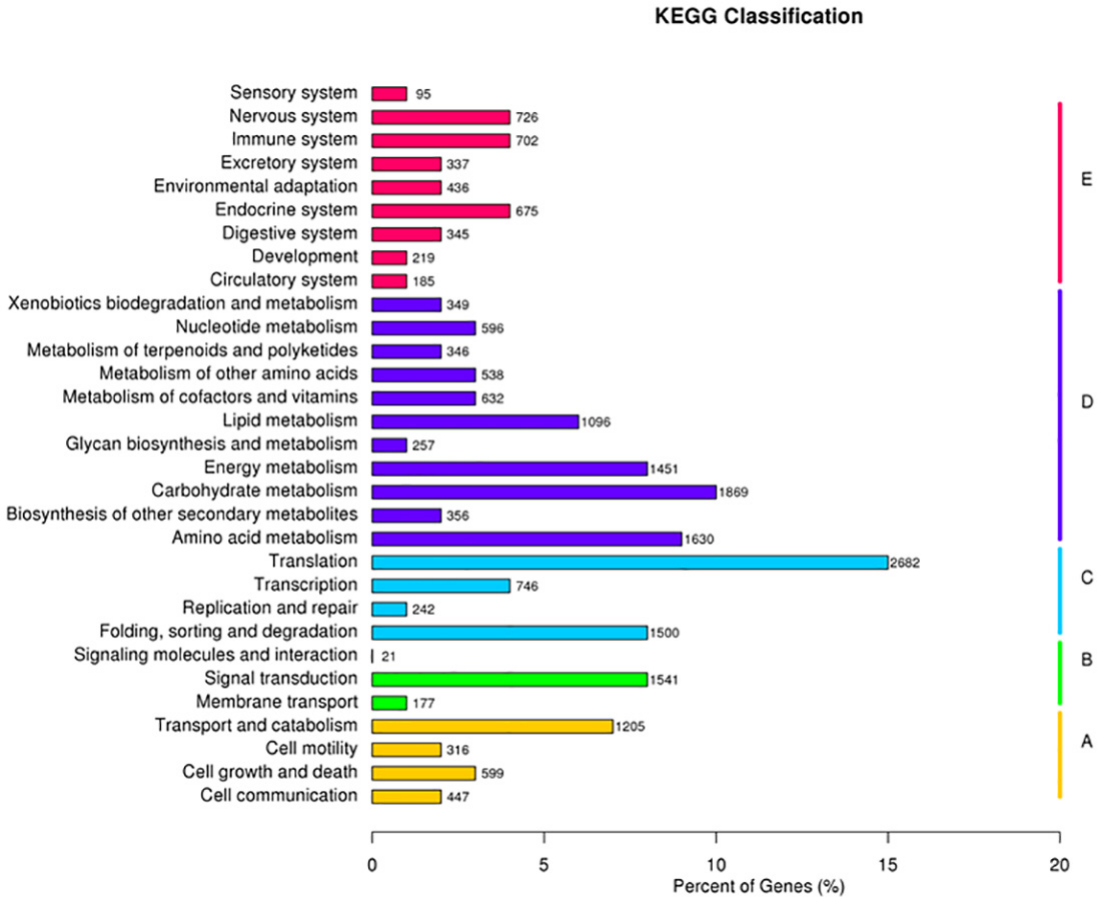
Pathway assignment based on Kyoto Encyclopedia of Genes and Genomes (KEGG) analysis. Classification based on the (A) cellular processes categories, (B) environmental information processing categories, (C) genetic information processing categories, (D) metabolism categories, (E) the organismal systems categories.

Kyoto Encyclopedia of Genes and Genomes (KEGG) analysis with a cutoff of E<1E-5 was used to further understanding of the transcriptome data. Of the 119,332 unigenes, 18,286 (13.96%) with significant matches from the database were assigned to 5 main categories, including 254 KEGG pathways (Table 2, S4 Table). As shown in Fig 3, among the 5 main categories, metabolism was the biggest category (9120, 49.87%), followed by genetic information (5170, 28.27%), organismal systems (3720, 20.34%), cellular processes (2567, 14.04%) and environmental information processing (1739, 9.51%) (Fig 3).

### Differentially Expressed Gene (DEG) Analysis among the Three Libraries

**Fig 4:**
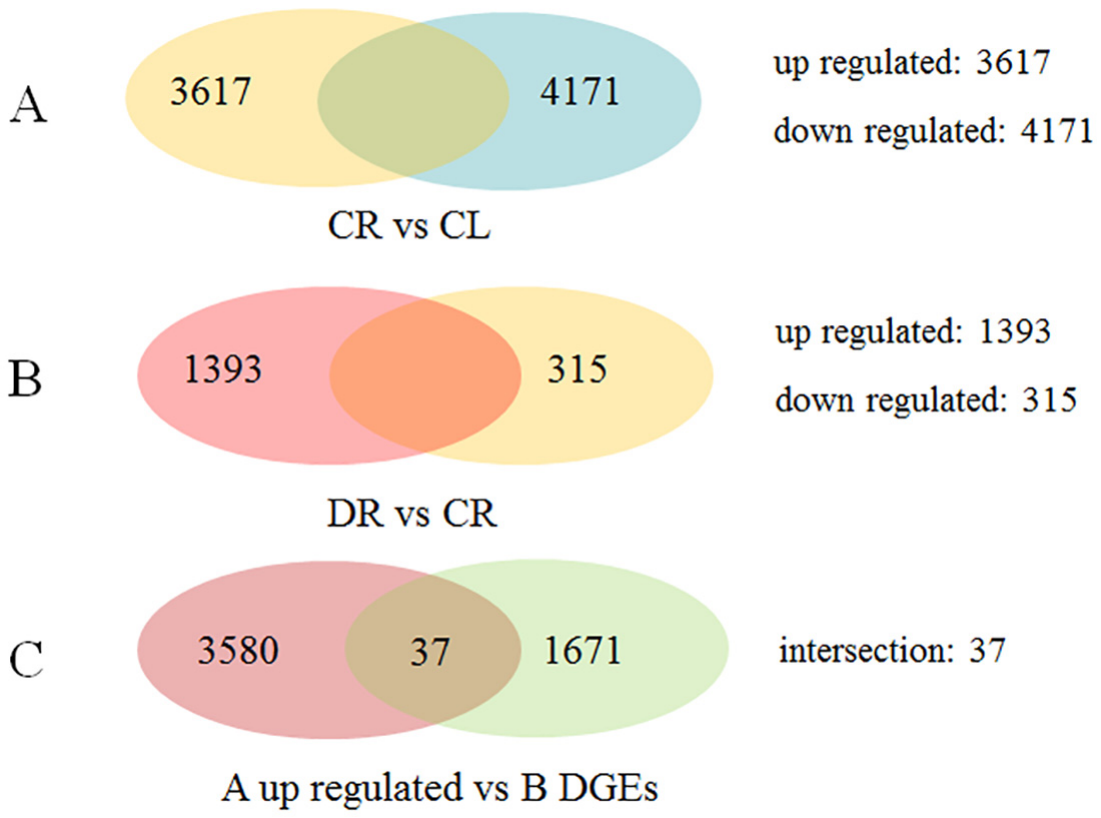
Three digital gene expression (DGE) libraries of the common wild rice. (A) DEGs obtained by comparing the CR and CL libraries. (B) DEGs obtained by comparing the CR and DR libraries. (C) DEGs identified from the up-regulated genes in A compared with DEGs in B.

To find the drought-induced genes in root, DEG analysis of the three transcriptome libraries was performed. First, comparing the CR and CL libraries showed that 3617 genes were up-regulated, and 4171 genes were down-regulated (Fig 4A, S5 Table). Among all DEGs, 535 were expressed in roots but not expressed in shoots (S5 Table).

According to the analysis of the differentially expressed genes between the roots (CR) and shoots (CL) shown in Fig 4A, Gene Ontology (GO) analysis on DEGs was performed based on the NR annotation. Among the 7788 DEGs, 4262 were assigned to 60 GO terms (S2A Fig), and of these genes, 2062 of the 3617 up-regulated genes and 2200 of the 4171 down-regulated genes were assigned to at least one of the GO terms(molecular function, cellular component, or biological process). Some GO terms, such as carbohydrate metabolic process, response to oxidative stress were enriched in both sets of transcripts from the two tissues. The results showed that there must be some same biological process for root activities and leaf photosynthesis. However, some significant differences were found between the two sets of enriched GO terms. In particular, GO terms related to photosynthesis and organelles were highly enriched in DEGs for roots and shoots, respectively, which also showed the expression of specific genes in different tissues. For the biological process category, 11 GO terms were found to be involved in responses to various stimuli (Table 4); response to stimulus was the most common (1364, 22.40%), followed by response to stress (757, 12.43%), response to chemical stimulus (408, 6.70%), response to abiotic stimulus (326, 5.35%), response to oxidative stress (156, 2.56%) and response to temperature stimulus (109, 1.79%) (Table 4).

**Table 4:** GO terms for the response to abiotic stimuli related to differentially expressed genes.

GO Term	Name Ontology	CR vs CL (DEG_ item)		Percentage (%)	*P*
		Up	Down		
GO:0006979	response to oxidative stress	90	66	2.56	1.2E-31
GO:0009628	response to abiotic stimulus	133	193	5.35	3.38E-28
GO:0006950	response to stress	411	346	12.43	2.94E-20
GO:0042221	response to chemical stimulus	197	211	6.70	7.45E-20
GO:0050896	response to stimulus	696	668	22.40	8.44E-20
GO:0009416	response to light stimulus	38	98	2.23	2.61E-19
GO:0009314	response to radiation	40	99	2.28	3.67E-19
GO:0010035	response to inorganic substance	87	101	3.09	4.95E-15
GO:0009266	response to temperature stimulus	41	68	1.79	1.06E-12
GO:0009409	response to cold	30	50	1.31	1.68E-11
GO:0010038	response to metal ion	66	62	2.10	1.08E-10

To demonstrate the degree of enrichment of the response-related genes, the 11 GO terms were analyzed by the TopGO analysis. This analysis found that degree of response to abiotic stimuli was enriched the most, followed by the response to oxidative stress (S2B Fig). Among the up-regulated genes in the CR library, the strongest up-regulation occurred for the responses to stress, response to abiotic and response to oxidative stress (S2 Fig). These results showed that gene expression responses were different in different tissues; that is, genes in common wild rice show specificity of expression. Meanwhile, the DR and CR libraries were also compared, which showed the up-regulation of 1393 genes and the down-regulation of 315 genes in the DR library compared with the CR library (Fig 4B, S6 Table).

Transcription factors (TFs) is a group of protein molecule that specifically bind to the 5'-end upstream sequence of targeted gene to ensure genes^’^ expression under specific temporal and spatial condition. On the other hand, transcription factors are associated with the regulation of plant drought stress responses. For example, DREB transcription factor is a dehydration-responsive elements binding protein which has important regulatory roles during drought, salt and cold stress responses in plants.

TFs analysis was performed based on the Swiss-Prot annotation. The present study found that the differentially expressed TF genes of belonged to a diverse range of TF families (Table 5). Numerous transcription factors showed differential expression between the CR and CL libraries, including 40 *MYB* genes, 31 *AP2/ERF* genes, 4 *HSF* genes, 5 *NAC* genes, and 21 *WRKY* genes, etc. The comparison between the DR and CR libraries showed that 4 *MYB* genes, 19 *Znf* genes and 2 *DREB* gene were up-regulated in roots after drought stress.

**Table 5:** Distribution of differentially expressed transcription factor families.

TF family	DR vs CR		CR vs CL	
	Up-regulated	Down-regulated	Up-regulated	Down-regulated
MYB	4	3	19	21
AP2/ERF	1	0	17	14
Znf (WRKY)	0	0	15	6
Znf (RING-finger)	2	2	20	16
Znf	19	5	104	103
NAC	0	2	4	1
bZIP	7	1	21	15
HSF	0	0	2	2
Aux/IAA	1	0	3	3
CBF	1	0	18	1
DREB	2	0	1	3

Each transcription factor contains known DNA-binding domains defined by the Pfam database.

Finally, the 3617 genes showing higher expression in the CR than CL library were compared with the 1708 genes showing differential expression between the DR and CR libraries. Thirty-seven genes were identified in both comparisons (Fig 4C, Table 6). That mean these genes were drought-induced and expressed specifically in root tissue. Of these 37 genes, 17 could not be matched to any genes in the database, that mean maybe these 17 genes were might be specific to the response to drought stress in common wild rice. Besides different types of transcriptional factors, some other types of genes have been discovered in this study, such as peroxidases. In this study 76 peroxidases related genes were found (S5 Table). Among the 76 genes, 48 genes were up-regulated in roots and 28 genes were up-regulated in leaves, meanwhile, 12 and 2 genes were found not expressing in leaves and roots, respectively. And 6 peroxidases genes were found in the DEGs between DR and CR (S6 Table).

**Table 6:** The thirty-seven differentially expressed genes identified from the three libraries.

Gene_id	PFAM description	BP Description	NR E-value
comp106888_c0	—	—	—
comp107776_c0	—	—	—
comp32290_c1	—	—	—
comp33316_c0	Alphaherpesvirus glycoprotein E	—	6.2E-11
comp44002_c0	Ribosomal protein L4/L1 family	ribosome biogenesis	3.7E-145
comp48589_c0	—	—	—
comp53838_c0	—	—	4.32E-11
comp56774_c0	ADP-ribosylation factor family	intracellular protein transport	2.3E-110
comp57306_c0	Myb-like DNA-binding domain	—	4.46E-34
comp60508_c0	Brevenin/esculentin/gaegurin/rugosin family	defense response	—
comp62152_c1	Tap, RNA-binding	protein phosphorylation	9.48E-92
comp63362_c0	—	—	—
comp65617_c0	—	—	—
comp66822_c0	PRONE (Plant-specific Rop nucleotide exchanger)	root hair cell development	0
comp70721_c2	Peroxidase	response to oxidative stress	2.6E-99
comp73162_c3	Cecropin family	—	5.53E-18
comp73227_c1	—	—	0
comp74521_c0	Tubulin C-terminal domain	protein polymerization	0
comp75305_c0	Myb-like DNA-binding domain	—	3.43E-75
comp75490_c0	—	—	—
comp75573_c1	Membrane transport protein	oxidation-reduction process	1.4E-115
comp75601_c0	—	—	—
comp76407_c5	EamA-like transporter family	—	3E-119
comp76878_c0	Type III restriction enzyme, res subunit	pathogenesis	0
comp77894_c0	—	—	3.6E-109
comp78054_c0	—	—	—
comp78880_c0	Peroxidase	response to oxidative stress	1.49E-41
comp79092_c0	Calponin homology (CH) domain	—	2.5E-146
comp81484_c0	Glycosyltransferase family 28 N-terminal domain	carbohydrate metabolic process	1.19E-43
comp83193_c0	Cytochrome b559, alpha (gene psbE) and beta (gene psbF) subunits	photosynthesis	—
comp83816_c0	—	—	—
comp84209_c0	Fz domain	—	—
comp84868_c0	—	—	—
comp84983_c0	Sarcoglycan alpha	—	—
comp87399_c0	—	—	—
comp88336_c0	—	—	—
comp97810_c0	—	—	1.4E-63

—, no hits in the specific database

### Verification of the Expression of Selected Differentially Regulated Genes by Quantitative Real-Time PCR

**Fig 5:**
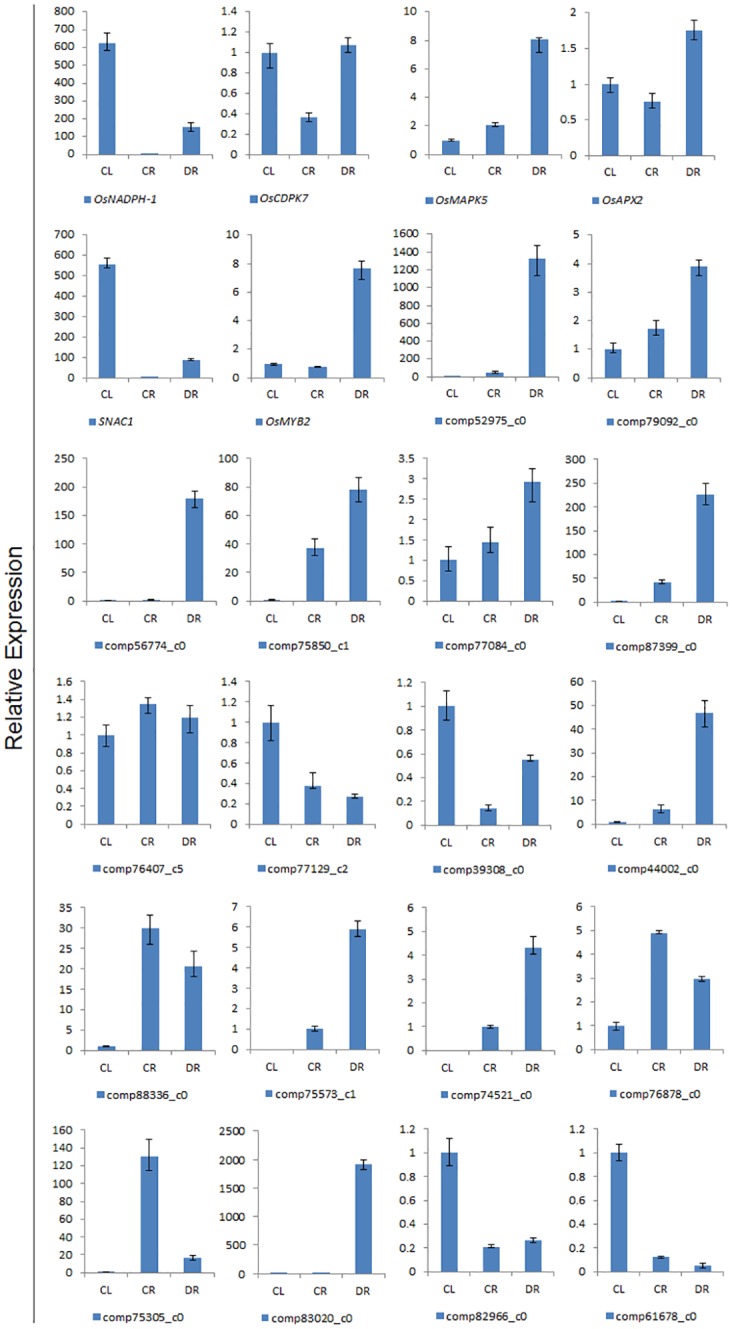
Confirmation of the transcriptomic profiles of selected genes by qRT-PCR. The x-axis shows the three samples; the expression level of CL was used as a control. The y-axis shows the relative quantitative expression level of each unigene.

To confirm the gene expression data, 18 up or down-regulated unigenes were randomly chosen from the three libraries for qRT-PCR analysis. Among the 18 genes, 10 genes belonged to the 37 selected DEGs. For most of the 18 genes, their expression patterns in the real-time PCR analysis were similar to those predicted except comp77129_c2 and comp39308_c0 by the transcriptome sequencing data, for example the comp75573_c1 and comp74521_c0 genes were not expressed in shoots (Fig 5). The expression level of six known drought response genes significantly increased after drought stress. These results were consistent with that of previous researches.

**Fig 6:**
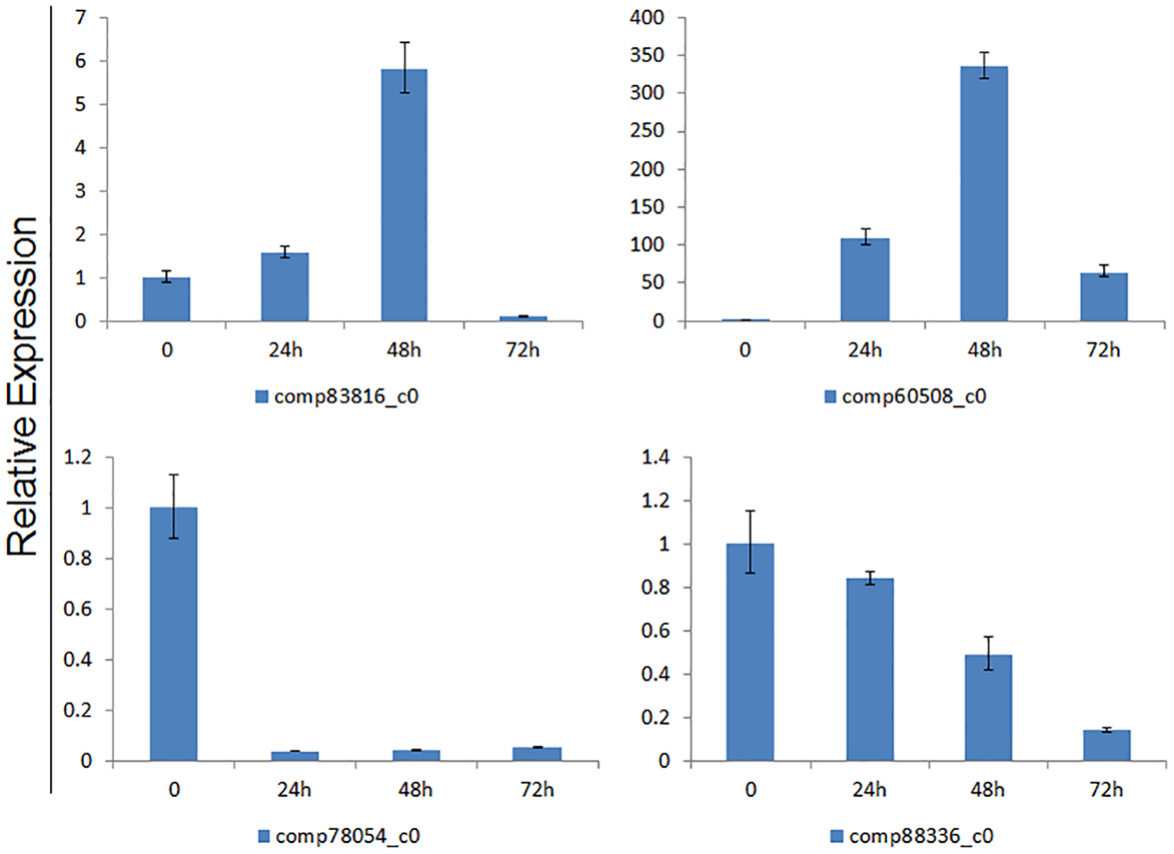
Expression characters of four selected unigenes in the roots of PEG-treated plants at 0 h, 24 h, 48 h and 72 h. The relative expression value was calculated using the 2^-ΔΔCt^ method and 0 h as a control sample.

Next, we tested the expression patterns of four of the 37 differentially expression genes to testing the expression pattern in response to drought stress (15% PEG-6000) in root tissues. The transcription sequencing results showed that two of these four genes, comp83816_c0 and comp60508_c0, gene were up regulated by drought, and the other two genes, comp78054_c0 and comp88336_c0, were down-regulated by drought. In the qRT-PCR experiment, the transcription levels of the comp83816_c0 and comp60508_c0 genes gradually increased in response to drought stress at 1 to 2 days and then decreased at 3 days. The transcript level of the comp88336_c0 gene gradually decreased in response to drought stress on days 1 to 3, and the level of comp88336_c0 quickly decreased in response to drought stress (Fig 6).

### Microsatellite Distribution Analysis and EST-SSR Marker Development

In order to analyze the microsatellites distribution in the common wild rice genome, the 119,332 unigenes generated in this study were used to mine potential microsatellites that were defined as mono- to hexanucleotide motifs with a minimum of three repetitions. A total of 26,574 potential simple sequence repeats (SSRs) were identified in 20,586 unigenes. Of these 20,586 unigenes, 15,973 and 4613 unigenes contained one or more than one SSR, respectively (Table 7). The number of potential EST-SSRs per unigene varied from 1 to 9, with an average of 1.29. For the motif types, the Tri-nucleotide type was the most one. Using the SSR-containing sequences, 12,324 SSR sites were designed EST-SSR primers with the Primer Premier 3.0 software. Information about these EST-SSR primers is shown in S7 Table. In the 12,324 SSR sites, about 2220 unigenes which had SSR sites were differentially express between CR and CL, while only 145 DEGs found between DR and CR.

**Table 7:** Summary of the EST-SSRs that were identified in the transcriptome data.

Search item	Numbers
Total number of examined unigenes	119,332
Total size of examined sequences (bp)	85,352,510
Total number of identified EST-SSRs	26,574
Number of EST-SSRs containing sequences	20,586
Number of sequences containing more than one SSR	4,613
Mono-nucleotide	9,889
Di-nucleotide	3,871
Tri-nucleotide	12,378
Tetra-nucleotide	392
Penta-nucleotide	29
Hexa-nucleotide	15

## Discussion

As one of the important component in wild rice, common wild rice is a very useful resource which has many useful genes could be used for cultivated rice improvement. Many genes associated with important agronomic traits, such as domestication, disease resistance, and insect resistance, have been identified [30, 33, 34, 44–46]. In previous studies researchers used common wild rice and cultivated rice to do hybridization and got many inbred lines to increase the drought resistance [35]. While until now there is little information about the drought-related genes resources in common wild rice. In the current study, we used next-generation sequencing technologies to analyze the transcriptomic changes that occur in common wild rice during drought stress to identify the genes that are induced in roots under this condition. As expected, we identified many known genes, including transcription factors, and some possible new genes to be involved in the drought-response pathway in common wild rice.

More than 33 million raw reads were generated by Illumina sequencing. With de novo assembly, the raw reads were further assembled into 119,332 unigenes, which was much higher than the number of genes in found in cultivated Japonica rice. One possible reason for this difference in gene number is the high heterozygosity of common wild rice [47,48]. The N50 length of the unigenes was 1147 bp, and the average length was 715 bp; these results were comparable to other recently published plant transcriptomic analyses, such as that for *Reaumuria soongorica* (N50 = 1109 bp, average length = 677 bp) [49]. More than half of the unigenes identified in this study (65856, 55.18%) were successfully assigned to annotated genes by BLAST against public databases. Among the unigenes, a BLAST against the nt database showed that 62,572 (52.40%) had significant homology to functional genes encoding specific proteins in *Oryza sativa* Japonica. As expected, most of the genes (64.7%) were matched to cultivated rice, indicating that the genome of cultivated rice is highly similar to that of common wild rice. However, other than the matched genes, no genes could be matched to any database, indicating that common wild rice contains some new and nonconserved genes. After unigene assembly, the DEGs were screened using comparative analyses of the CL, CR and DR libraries. When comparing the CL and CR libraries, 366 DEGs were found to be only expressed in leaves, and 535 were only expressed in roots, indicating that these genes were tissue-specific. This result was consistent with results in previous studies. Some tissue specific gene, such as root-specific gene *OsEXPA8* and cell-wall invertase genes of rice at flowering have been identified [50,51]. Furthermore, the gene expression patterns of different organs of rice shoots under the drought and high-salinity stress have also been revealed [52]. Many of the tissue-specific genes identified in this study were associated with abiotic stresses, including water, cold, and heat stress (S8 Table), suggesting that common wild rice tissue has a unique strategy for managing environmental stress.

Previous researches have shown that gene expression altering when plants exposed to drought stress. To discover more drought-related genes that are specifically expressed in roots, we enlarged the threshold value to P-value<0.1. After performing DEG analysis, 37 possible drought-related and tissue-specific genes were identified, including some known transcriptional factors, such as the MYB genes [53].

After analyzing the DEG information, we selected six drought related genes, including one perpxidases gene to do real-time PCR to check the expression character during the drought treatment (Fig 6). The differentially expressed genes between CR and CL showed that this type of gene has the character of tissue specific in plants, and the differentially expressed genes between DR and CR showed that the increasing expression level of perpxidases genes could enhance the peroxisome level in cells to protect the cells in plants.

Among the 37 DEGs regulated by drought stress in common wild rice, over 50% had no homologs in the NCBI database, indicating that some of these genes may represent new drought-related transcripts that have not been reported in plants and that these genes may be useful resources for the genetic improvement of crops.

Roots and leaves are important tissues which could percept the drought stress information. In this study, we didn’t analyze the sequence data from drought treated leaves as we want to extract the important information from roots, and the gene information from drought leaves will be further analyzed in the future.

## Supporting Information

S1 Fig Transcripts and unigenes of assembly length distributions the proportion. The lengths of the interval assembly transcripts/unigenes are plotted on the horizontal axis, and the lengths of each assembled transcript are plotted on the vertical axis.
(TIF)

S2 Fig Gene Ontology enrichment and topGO analysis of the differentially expressed unigenes (CR vs CL). (A) Gene Ontology (GO) categorization of the DEGs. (B) DAG graph of the topGO analysis for the DEGs. (C) DAG graph of the topGO analysis for the DEGs that are up-regulated by drought. Each cycle or square represents one GO term. The more dark colour showed that the gene enrichment density was higher.
(TIF)

S1 Table Summary of Gene-specific primers used in the qPCR analysis are listed and expression levels in the library. (XLSX)

S2 Table Blast file for gene annotation. (XLS)

S3 Table Summary of GO classification of assembled unigenes. (XLSX)

S4 Table Summary of KEGG classification of assembled unigenes. (XLSX)

S5 Table Summary of annotation of differentially expressed (CRvsCL). (XLSX)

S6 Table Summary of annotation of differentially expressed genes(DRvsCR). (XLSX)

S7 Table EST-SSR primers and the contained unigene information. (XLSX)

S8 Table Summary of Response to stress related genes of differentially expressed. (XLSX)
