# Supplementary figures and images for: De novo Transcriptome Assembly of Common Wild Rice (Oryza rufipogon Griff.) and Discovery of Drought-Response Genes in Root Tissue Based on Transcriptomic Data
Source: PLoS One. 2015 Jul 2;10(7):e0131455. doi: 10.1371/journal.pone.0131455 (PMC4489613; doi:10.1371/journal.pone.0131455)

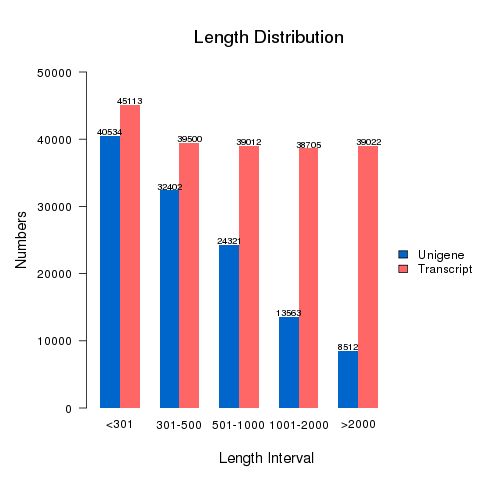

Supplement: S1 Fig — The lengths of the interval assembly transcripts/unigenes are plotted on the horizontal axis, and the lengths of each assembled transcript are plotted on the vertical axis. (TIF) [file pone.0131455.s001.tif]

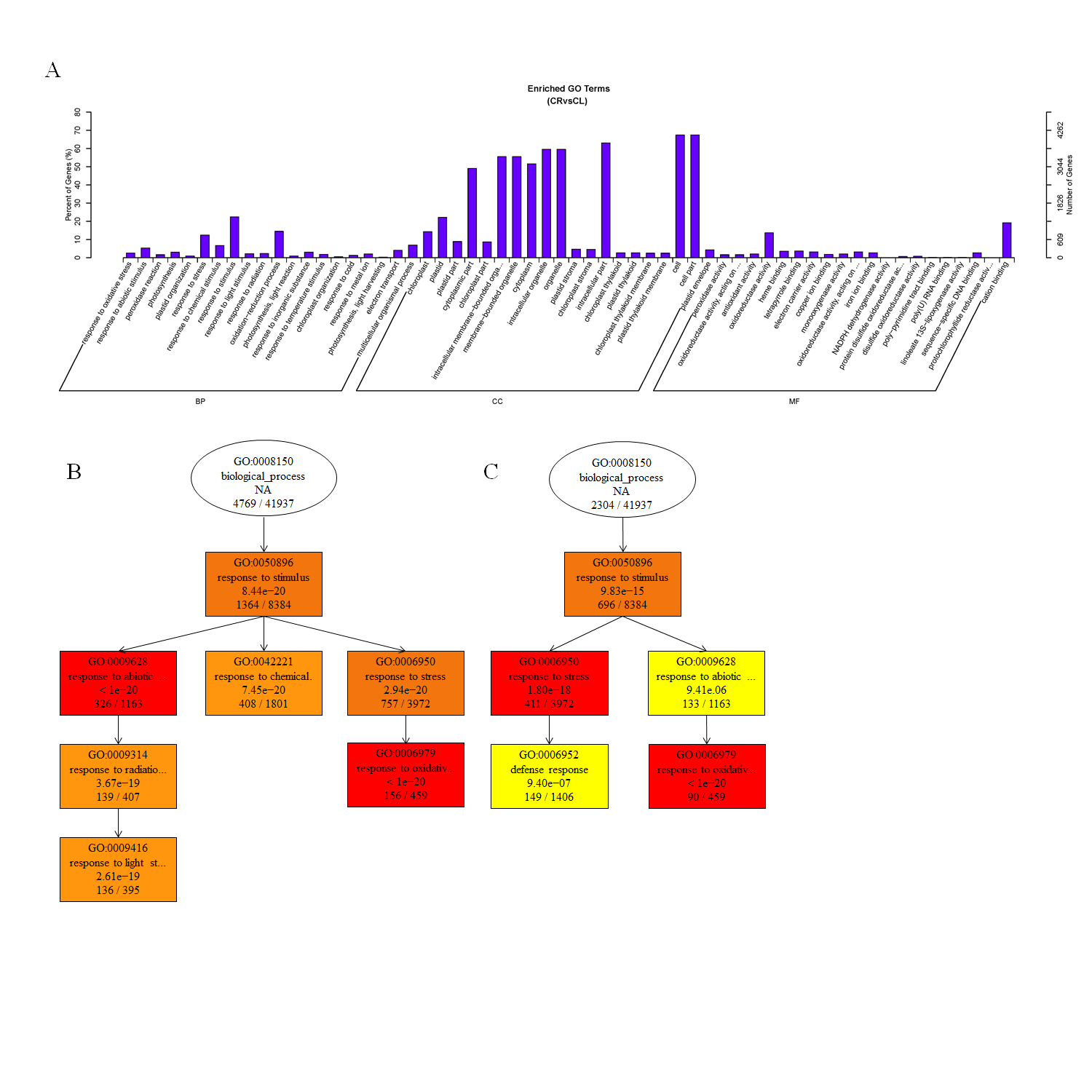

Supplement: S2 Fig — (A) Gene Ontology (GO) categorization of the DEGs. (B) DAG graph of the topGO analysis for the DEGs. (C) DAG graph of the topGO analysis for the DEGs that are up-regulated by drought. Each cycle or square represents one GO term. The more dark colour showed that the gene enrichment density was higher. (TIF) [file pone.0131455.s002.tif]
